# Guanidine Affects Differentially the Twitch Response of Diaphragm, Extensor Digitorum Longus and Soleus Nerve-Muscle Preparations of Mice

**DOI:** 10.3390/molecules17067503

**Published:** 2012-06-15

**Authors:** Rosana Ferrari, Léa Rodrigues-Simioni, Maria Alice da Cruz Höfling

**Affiliations:** 1 Department of Histology and Embryology, Institute of Biology, University of Campinas (UNICAMP), P.O. Box 6109, 13 083-970, Campinas, SP, Brazil; Email: ro.ferrari74@hotmail.com; 2 Department of Biology, Institute of Biosciences, São Paulo State University (UNESP), Av. 24A, no.1515, Bela Vista, CEP 13506-900, Rio Claro, São Paulo, Brazil; 3 Department of Pharmacology, Faculty of Medical Sciences, University of Campinas (UNICAMP), P.O. Box 6111, 13 083-970, Campinas, SP, Brazil; Email: simioni@unicamp.br

**Keywords:** neuromuscular transmission, transmitter release, contractility, skeletal muscle

## Abstract

Guanidine has been used with some success to treat myasthenia gravis and myasthenic syndrome because it increases acetylcholine release at nerve terminals through K^+^, Na^+^ and Ca^2+^ channels-involving mechanisms. Currently, guanidine derivatives have been proposed for treatment of several diseases. Studies aimed at providing new insights to the drug are relevant. Experimentally, guanidine (10 mM) induces on mouse phrenic nerve-diaphragm (PND) preparations neurotransmission facilitation followed by blockade and a greatest secondary facilitation after its removal from bath. Herein, we hypothesized that this peculiar triphasic response may differ in muscles with distinct twitch/metabolic characteristics. Morphological alterations and contractile response of PND, *extensor digitorum longus* (EDL) and *soleus* (SOL) preparations incubated with guanidine (10 mM) for 15, 30, 60 min were analyzed. Guanidine concentrations of 5 mM (for PND and EDL) and 1 mM (for EDL) were also tested. Guanidine triphasic effect was only observed on PND regardless the concentration. The morphological alterations in muscle tissue varied along time but did not impede the PND post-wash facilitation. Higher doses (20–25 mM) did not increase EDL or SOL neurotransmission. The data suggest a complex mechanism likely dependent on the metabolic/contractile muscle phenotype; muscle fiber types and density/type of ion channels, sarcoplasmic reticulum and mitochondria organization may have profound impact on the levels and isoform expression pattern of Ca^2+^ regulatory membrane proteins so reflecting regulation of calcium handling and contractile response in different types of muscle.

## 1. Introduction

The contractile response of skeletal muscle can be affected by a variety of substances which can act directly either on the neurotransmission or contractile apparatus, inhibiting and/or facilitating the muscular response. Some of these substances are from animal [1,2,3,4] or plant origin [5,6,7]; others are synthetic products such as tetraethylammonium, aminopyridines and guanidine. All of these substances have been used as tools to improve our understanding on the mechanisms of functioning of neuromuscular junction (NMJ). The highly specific action of these substances allowed them to mimic physiological and pathophysiological events related to muscle contractility, advancing in the knowledge of ion channels and contractile machinery function/dysfunction during excitation-contraction (EC) coupling. For instance, tetraethylammonium (TEA) or 3,4-diaminopyridine (3,4-DAP) increases action potential duration in nerve terminals through the blockade of K^+^ channels, resulting in increased Ca^2+^ influx into the terminal and release of acetylcholine (ACh) [8,9,10]. The prolongation of the presynaptic action potential caused by K^+^ channel blockade and further activation of voltage-dependent Ca^2+^ channels at nerve terminals are also properties displayed by guanidine [11]. TEA, 3,4-DAP and guanidine antagonize the neuromuscular paralysis produced by botulinum toxin [11]. The guanidine molecule [HN=C(NH_2_)_2_] can also reverse the neuromuscular blocking effect of dibekacin and d-tubocurarine [12,13]. The intensity of these effects has been shown to be influenced by Ca^2+^ intracellular concentration and would result mainly from blockade of fast K^+^ channels, and then modulation of Ca^2+^ entry into nerve terminals [14]. In the past, guanidine was used with some success to treat myasthenia gravis [15] and myasthenic syndrome [16] because of its ability to improve the neuromuscular transmission, as first described by Feng [17,18]. Guanidine was also shown to be protective against gramicidin toxicity in NG108-15 (neuroblastoma x glioma) hybrid cells [19]. Recently, guanidine derivatives have been proposed for therapeutic use in neuromuscular disorders [20], indicating the drug potential as a research tool in neurobiology. Experimentally, we have reproduced the facilitation of ACh release by guanidine in the chick biventer cervicis and mouse phrenic nerve-diaphragm (PND) preparations; interestingly, a peculiar triphasic effect characterized by an initial facilitation followed by a total neuromuscular blockade and then a secondary facilitation, with amplitude much greater than the former, was evoked when guanidine (10 mM) was removed from bath by washings [21]. In addition, studies with light and transmission electron microscopy showed that a number of fibers showed swelling and rupture of mitochondria and sarcoplasmic reticulum (SR), and alterations in pre- and postsynaptic structures of the NMJ [22]. Other fibers remained morphologically normal. These changes are compatible with ionic unbalance and in line with the inhibitory effect of guanidine on K^+^ and Ca^2+^ channels and Na^+^ conductance. Such morphological disturbances were only partially prevented by tetrodotoxin (TTX) preincubation. Since the triphasic effect of guanidine on PND was fully accomplished after 60 min of incubation [21], it is clear that the alterations produced on mitochondria, SR and pre- and postsynaptic components [22] did not prevent the EC coupling machinery to promote the post-washing marked secondary facilitation. Whether the triphasic effect could occur in earliest periods of incubation, such as 15 and 30 min, corresponding to primary facilitation and reduction phase of the contractile response, respectively, has not been investigated so far. Likewise, the proportion of morphological changes at these earliest periods when compared with those observed after 60 min of incubation is unknown. Would the extension of damage have correlation with the contractile response? In addition, could the triphasic effect and proportion of muscle damage seen in the mouse diaphragm be equally evoked by guanidine in peripheral (limb) muscle preparations? 

The diaphragm is a muscle with distinctive physiological properties when compared with peripheral muscles; it is resistant to the blocking effects of competitive neuromuscular blocking agents due to the high concentration of quanta of ACh released after stimulation and greater number of occupied postsynaptic nicotinic receptors [23]; along with a low acetylcholinesterase activity at the synaptic cleft [24] and a major quantal content when compared, for example, with EDL [23]. Moreover, the diaphragm muscle comprises motor units varying in their mechanical and fatigue properties and which rely on the fiber type motor innervation control [25]. Interestingly, the decline of quantal content was shown to vary across NMJs of different muscle fiber types during repetitive stimulation, indicating that the underlying mechanism of synaptic delivery is in line with their contractile characteristics [26]. Since fast and slow twitch muscle fibers have distinct contractile properties (see [27] for review), it is not surprising that muscles with different proportion of these fiber types may respond differently to substances affecting neurotransmission. 

Herein, we hypothesized that the neuromuscular response to guanidine may differ in muscles with distinct twitch/metabolic characteristics, *i.e.*, the proportion of fibers with predominance of fast-twitch and low-twitch fibers and glycolytic and oxidative metabolism. The contractile response and morphology of phrenic nerve-diaphragm (PND), *extensor digitorum longus* (EDL) and *soleus* (SOL) preparations were investigated for 15, 30 and 60 min of guanidine incubation. The discussion of the results considered the characteristics of each muscle and the pharmacological/ultrastructural effects already described for guanidine on diaphragm after 60 min incubation. The elucidation of these issues will contribute to shed light on the guanidine pharmacological interaction with phenotypically different skeletal muscles and hence their neurotransmission mechanisms. A variety of pathophysiological disturbances are related to the dysfunction of K^+^ channel and Na^+^ channel activity and calcium homeostasis [28,29]. The use of depolarizing drugs affecting K^+^, Na^+^ and Ca^2+^ channels-dependent cellular events can be excellent tools for determining cell mechanisms, which results in either therapeutic benefits or undesirable side-effects. 

## 2. Results and Discussion

### 2.1. Myographic Recording

**Figure 1:**
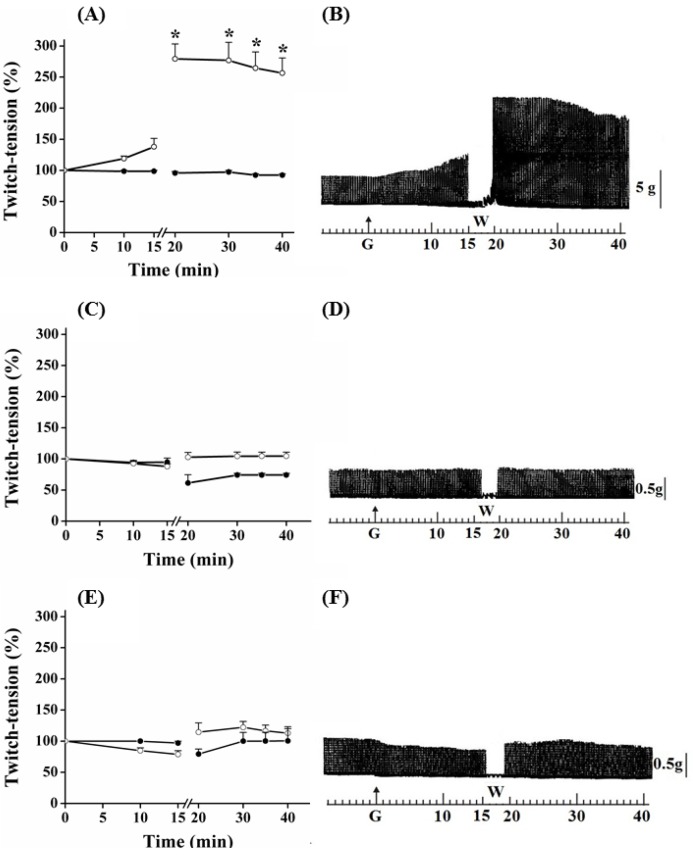
Effect of guanidine (10 mM) (white circles) for 15 min on the twitch response of PND (A), EDL (C) and SOL (E) (n = 8/each) in comparison with matched controls (black circles) (n = 3, 8, 8, respectively). The line breaking corresponds to the preparation washing (W). Each point represents the mean ± S.E.M; one-way ANOVA followed by Tukey test, * *P* ≤ 0.05. Panels B (PND), D (EDL) and F (SOL) display the myographic isometric twitch record of preparations incubated with guanidine (G). The vertical bars (5, 0.5 g) represent muscle tension.

**Figure 2:**
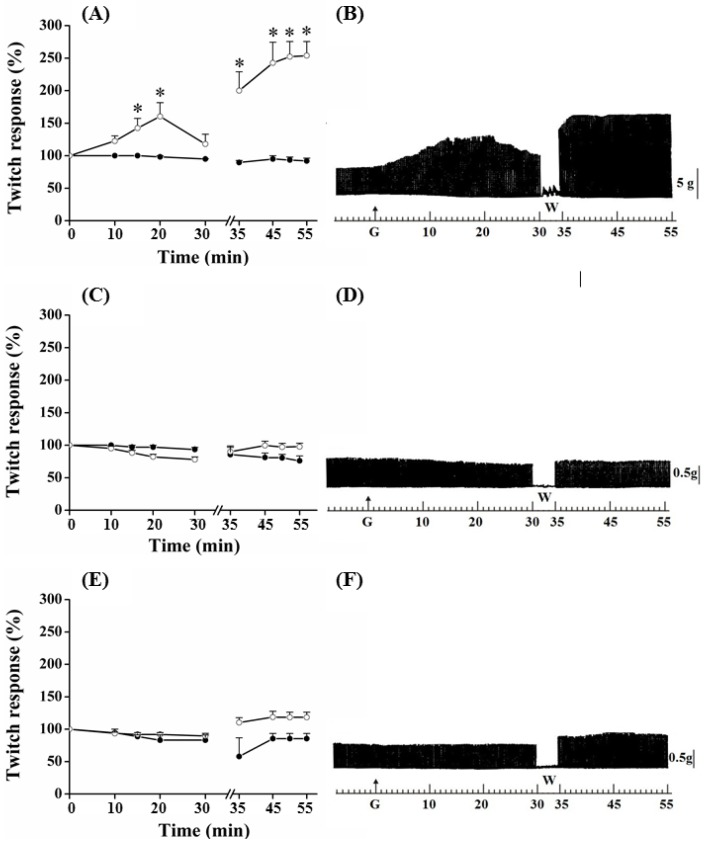
Effect of guanidine (10 mM) (white circles) for 30 min on the twitch response of the PND (A), EDL (C) and SOL (E) (n = 8/each) in comparison with matched controls (black circles) (n = 3, 8, 8, respectively). The line breaking corresponds to the preparation washing (W). Each point represents the mean ± S.E.M; one-way ANOVA followed by Tukey test, * *P* ≤ 0.05. Myographic isometric twitch records of PND (B), EDL (D) and SOL (F) incubated with guanidine (G). The vertical bars (5, 0.5 g) correspond to muscle tension.

**Figure 3:**
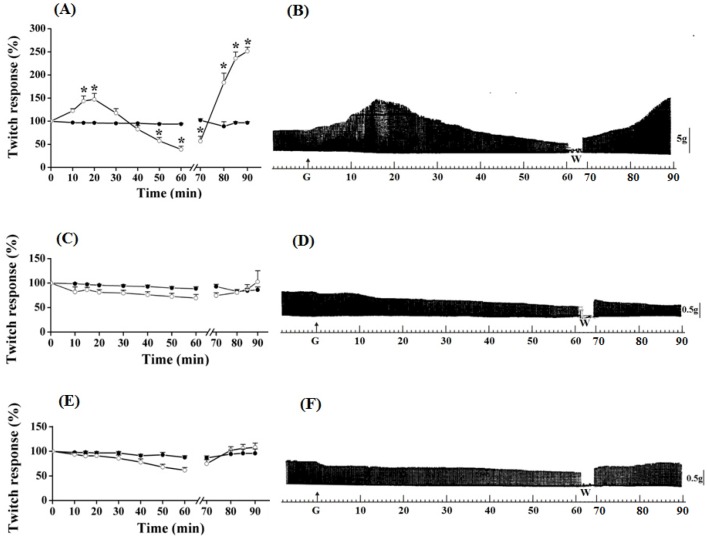
Effect of guanidine (10 mM) (white circles) for 60 min on twitch responses of PND (A), EDL (C) and SOL (E) (n = 8/each) in comparison with matched controls (black circles) (n = 3, 8, 8, respectively). The line breaking corresponds to the preparation washing (W). Each point represents the mean ± S.E.M; one-way ANOVA followed by Tukey test, * *P* ≤ 0.05. Myographic isometric twitch records of PND (B), EDL (D) and SOL (F) incubated with guanidine (G). The vertical bars (5, 0.5 g) correspond to muscle tension.

Myographic recordings of PND, EDL and SOL preparations incubated with guanidine for 15, 30 and 60 min, respectively are shown in Figure 1, Figure 2 and Figure 3. In PND preparation, guanidine (10 mM) determined a gradual neurotransmission facilitation which achieved a 35% increase after 15 min (*P* ≤ 0.05). The removal of guanidine from bath led to a second phase of facilitation of the contractile response, which was immediate rather than gradual, and almost four times (~170%) the amplitude of the initial record. Twenty minutes after guanidine removal by washing the preparation, the increase in the contractile response still remained 150% above that observed before guanidine administration and in relation to that presented by preparations incubated with Tyrode only (Figure 1A,B). 

Guanidine (10 mM) did not promote facilitation of neurotransmission in EDL and SOL preparations after 15 min of incubation (Figure 1C,D and 1E,F, respectively). On the contrary, both tended to have reduced responses, which was more evident in SOL. Guanidine washing caused a nonsignificant tendency to increase the contractile response up to the remaining 20 min of observation. Likewise, neither 1 mM nor 5 mM guanidine promoted facilitation in EDL preparation even after its removal by washing (not shown).

PND contractile response, after the addition of guanidine (10 mM) in the bath for 30 min, is shown in Figure 2. In these preparations, guanidine induced a gradual increase in the neuromuscular activity, which peak reached 65% of increase at 20 min; thereafter, there was facilitation reduction, although it has remained approximately 20% above baseline at the end of 30 min. Removal of guanidine and replacement for Tyrode caused immediate facilitation in a proportion of 95% (almost twice the initial, primary, facilitation). 

The amplitude of the contractile response increased during the next 15 min, after which it remained stable; however, it was approximately 150% superior when comparing with baseline values (Figure 2A,B) (*P* ≤ 0.05). Guanidine (30 min) to the EDL preparation (Figure 2C,D) depressed not significantly the muscle contractile activity, whereas it caused no change to SOL (Figure 2E,F); the posterior tendency towards a secondary facilitation after guanidine removal from bath was not statistically significant. Neither 1 mM nor 5 mM altered the EDL contractile amplitude (not shown).

The triphasic effect (primary facilitation/blockade/secondary facilitation of neurotransmission) of guanidine on motor response was selective for PND preparation (Figure 3). A gradual increase in the contraction amplitude was carried out, which peak (45–50% increase) occurred between 15 and 20 min. In sequence, it gradually decreased leading to total blockade within approximately 40 min and reaching negative values, approximately 70% under the baseline value, at 60 min (*P* ≤ 0.05).

**Figure 4:**
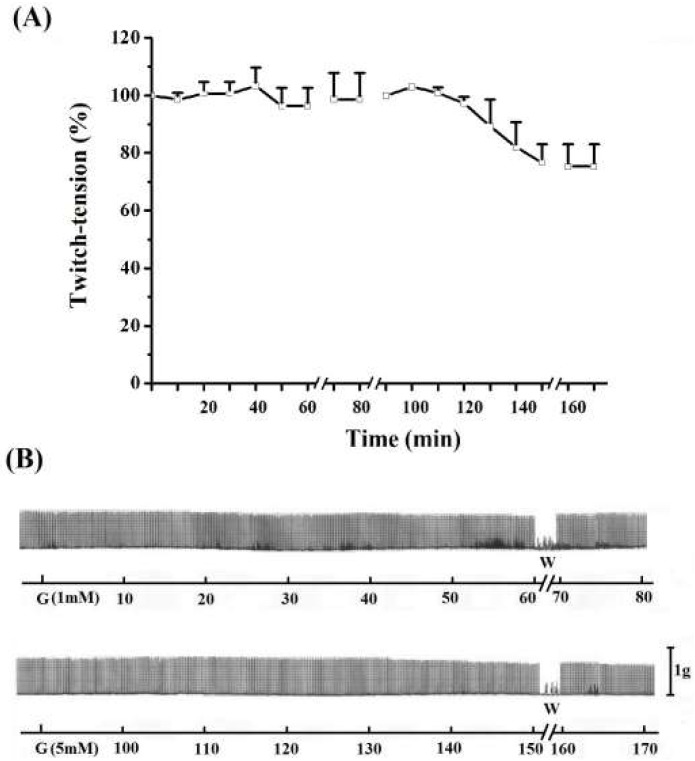
Effect of 1 mM (A) and 5 mM (B)guanidine (G) for 60 min on the twitch response of EDL (n = 3/each). The line breaking corresponds to the preparation washing (W). Each point represents the mean ± S.E.M; one-way ANOVA followed by Tukey test, * *P* ≤ 0.05. Panel B displays the myographic isometric twitch record of these preparations. The vertical bar (1 g) represents muscle tension.

In the diaphragm, the guanidine washout from bathing medium promoted a gradual reversal of neuromuscular blockade, reaching ~150% of facilitation (secondary) above baseline at 20 min (Figure 3A,B) (*P* ≤ 0.05). The secondary facilitation can be explained by the ability of guanidine to cross the Na^+^ channel [30,31], and this reduced the miniature endplate potentials (MEPPs) after washing preparations prior incubation with TTX [21]. Neither the primary nor the secondary facilitation was observed in EDL (Figure 3C,D) and SOL (Figure 3E,F) preparations incubated with guanidine for 60 min. The absence of primary and second facilitations in EDL (10 mM, 5 mM or 1 mM, see Figure 1, Figure 2, Figure 3 and Figure 4) and SOL (10 mM, Figure 1, Figure 2 and Figure 3) indicates that the triphasic effect of guanidine on neurotransmission occurs by a complex mechanism of action, depending on intrinsic muscle phenotypic and molecular characteristics. 

In addition, the effect of 5 mM guanidine was investigated by pre- or post-incubation with 4-aminopyridine (4-AP) in order to check either PND response before or after the blockade of potassium channels. Incubation of PND with 4-AP (10 µg/mL) produced as expected, a rise in the twitch response. The removal of 4-AP from bath by washing returned the twitch amplitude to baseline level. The subsequent addition of 5 mM guanidine produced facilitation of neurotransmission. After 80 min incubation with this low concentration of guanidine there was no blockade or inhibition of the amplitude of twitch response as was usual when 10 mM was used. Instead, the facilitation was maintained. After another washing for guanidine removal from bath the secondary facilitation did occur, almost achieving the level promoted by 4-AP (figure not shown). It was of note that there is a delay (which was variable in time) for starting the neurotransmission facilitation induced by guanidine after the 4-AP incubation and first washing.

**Figure 5:**
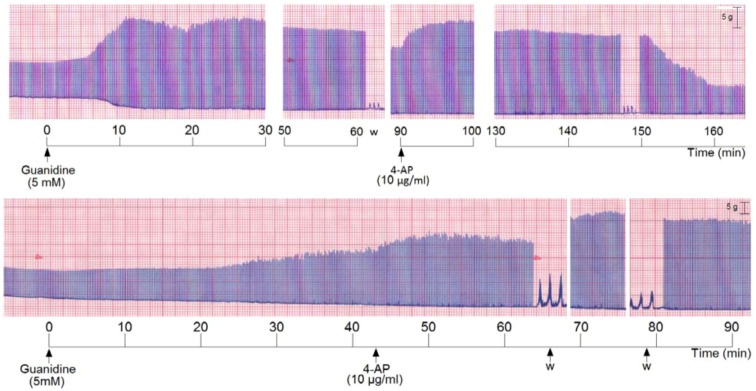
Representative recordings (of four experiments each) from indirectly stimulated PND incubated with 5 mM guanidine, followed by washing (w) and subsequent addition of 4-aminopiridinte (4-AP) and another washing after 60 min additional incubation (upper recording); in the lower recording there was no guanidine removal by washing before 4-AP addition.

We also incubated PND with 5 mM guanidine, then the preparation was washed and 4-AP was added. The addition of guanidine promoted a marked primary facilitation whose amplitude was reduced after 70–80 min. The subsequent washing and addition of 4-AP promoted another facilitation of neurotransmission which was maintained for the subsequent 60 min. The removal of 4-AP from bath by washing the preparation (three times) promoted an almost immediate decrease of the amplitude of the muscle contraction (Figure 5, upper register). A variation of these experiments was done which consisted in incubation of PND with 5 mM guanidine and after 45 min addition of 4-AP (10 µg/mL) without pre-washing. The 4-AP promoted an immediate rise in the twitch amplitude (~20%) which persisted until preparation was washed. The washing promoted a secondary facilitation and a third facilitation after the second washing of the preparation. The findings indicate that since guanidine was internalized into the fiber (by traversing sodium channels), the 4-AP pre-treatment did not interfere with the guanidine typical effect in diaphragm preparation (Figure 5, lower register). 

Taken together, the findings show that the blockade of K^+^ channels by 4-AP did not prevent the triphasisc effect characteristic of guanidine. In other words, 4-AP does not interfere with the primary and secondary increase of ACh release by guanidine (even with half of the concentration previously used). The findings suggest that even though both the 4-AP and guanidine are potassium channel blockers, guanidine exhibits some peculiar mechanisms not found in 4-AP. Interestingly when 4-AP is added without previous removal of the guanidine from preparation, there occurs two other facilitatory effects, after 4-AP removal by washing, in addition to the primary one.

Although several authors have suggested that guanidine effect relies on its action on voltage-gated Na^+^, K^+^ and Ca^2+^ channels, present either in the nerve terminal or skeletal muscle, its mechanism of action remains still not totally elucidated [11,21,22,30,32,33,34,35,36,37]. The time-dependent facilitatory effect of guanidine seen here in the diaphragm could be probably related to presynaptic action of the drug, increasing Ca^2+^ influx and enhancing release of ACh by nerve impulse [21,32], consequent to the blockade of K^+^ conductance in the nerve terminal, as well as increasing the duration of the presynaptic action potential [32,33,34,35,36].

The type of stimulation (indirect or direct field stimulation) and/or the guanidine concentration were/was responsible for differences in contractile responses between PND and EDL/SOL preparations seeming unlikely. PND even with direct stimulation responded with the typical triphasic effect (data not shown), and incubation with low or high doses of guanidine (5 mM, 20 or 25 mM) did not cause the triphasic effect on EDL and SOL preparations even after 60 min (data not shown). 

### 2.2. Morphology and Morphometry

**Figure 6:**
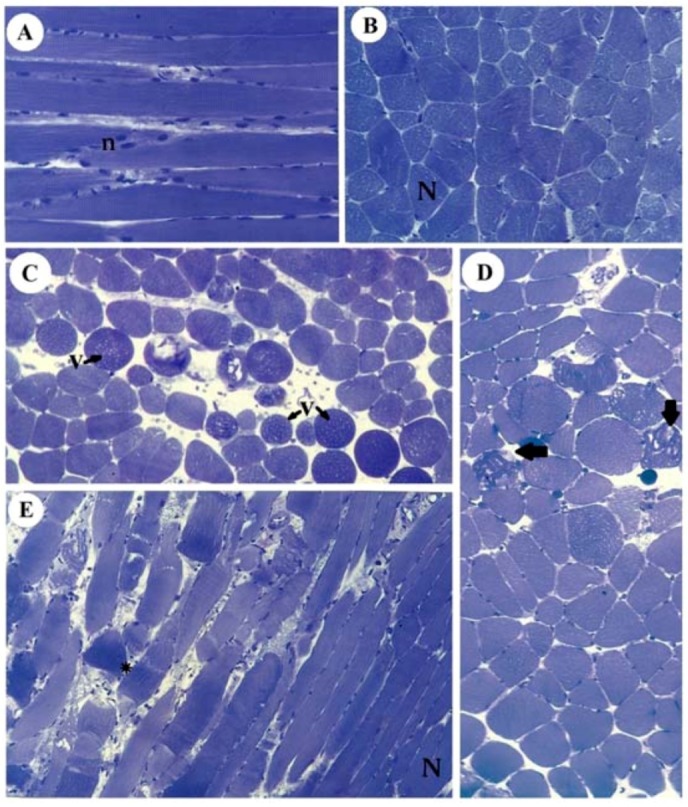
Light micrographs of PND incubated with Tyrode show normal fibers (N) with their well-positioned nuclei (n) (A: longitudinal section, B: transversal section) and of SOL (C), EDL (D) and PND (E) showing different stages of cellular disorganization after incubation with 10 mM guanidine for 60 min, under indirect electrical stimulation; altered cells show vacuolation (V), worm-like threads of myofibrils interspersed among empty- looking regions of sarcoplasm due to sarcolemma disruption (arrows) or hypercontraction caused by densely packed myofibrils (*). See also edematous (round in shape) and non-homogeneously stained myofibers (toluidine blue). Bar: 35 µm.

Figure 6 illustrates the normal aspect of muscle fibers of diaphragm incubated with Tyrode (panels A,B) and their different pathological stages in SOL (panel C), EDL (panel D) and diaphragm (panel E) after incubation with 10 mM guanidine for 60 min. Guanidine major effect was the appearance of vacuolated cells in SOL and EDL, which could be due to SR cisternae swelling, as reported for diaphragm [22]. 

**Figure 7:**
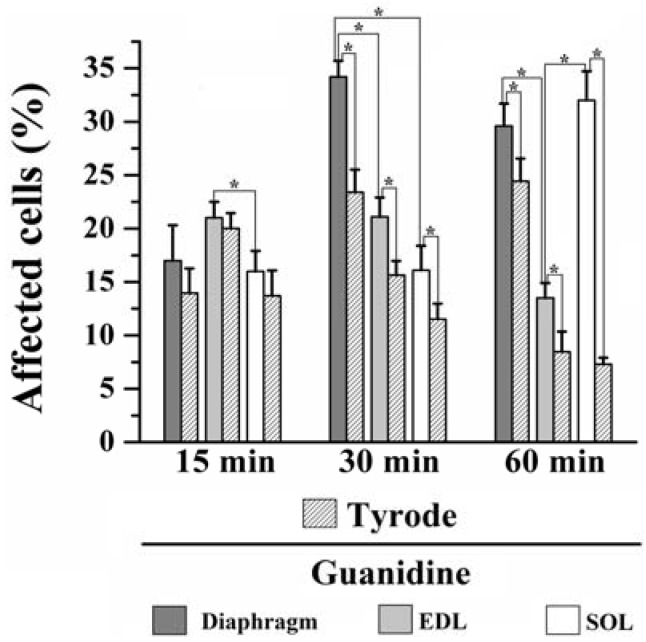
Proportion of affected cells in PND, EDL and SOL muscles after 15, 30 and 60 min of 10 mM guanidine incubation or Tyrode solution (control). One-way analysis of variance (ANOVA) followed by Tukey test, * *P* ≤ 0.05.

Moreover, instead of a typical polygonal profile in transverse sections, a number of fibers in all three preparations incubated with the organic cation guanidine showed spherical outline characteristic of swollen fibers; only a relative minor number of myofibers presented advanced pathological stages with color change due to condensed tortuous bands of myofibrils, or with clear spaces indicative of myofilament loss and sarcolemma rupture. The proportion of affected fibers caused by guanidine and Tyrode incubation is illustrated in Figure 7. 

*15 minutes incubation*: relative to Tyrode-incubated preparations, guanidine did not induce morphological alterations in PND, EDL or SOL (13% *vs.* 17% for PND; 20% *vs.* 21% for EDL and 13% *vs.* 16% for SOL of affected fibers). However, guanidine affected significantly more the EDL than SOL when compared to each other (*P* ≤ 0.05).

*30 minutes incubation*: relative to control, guanidine promoted a significant increase in the number of affected fibers in all three muscles compared to paired-controls (23% *vs.* 33% for PND; 15% *vs.* 21% for EDL and 11% *vs.* 16% for SOL), (*P* ≤ 0.05). In addition, there was significant difference when comparing the impact of guanidine effect in all three muscles which was highest for PND and lowest for SOL (*P* ≤ 0.05).

*60 min incubation: *compared to control, guanidine promoted a significant increase in the proportion of fibers damaged in all three muscles (24% *vs.* 28% for PND, 8% *vs.* 13% for EDL and 7.5% *vs.* 31% for SOL), (*P* ≤ 0.05). After 60 min, PND and SOL showed higher proportion of damage than EDL (P ≤ 0.05).

The findings give evidences that *in vitro* incubation with Tyrode solution also promoted fiber morphological alterations. Interestingly, for PND the higher the period of incubation (15, 30 and 60 min) the higher the proportions of fibers affected (13%, 23% and 24%, respectively), whilst for EDL and SOL the higher the time of incubation the lower proportion of fibers affected (20%, 15% and 8% for EDL and 13%, 11% and 7.5% for SOL). Taken the findings together, it seems that the interaction of *in vitro* condition and guanidine effect may give the relative injury caused by guanidine itself. The changes in the proportion of fiber damage may result, at least partially, from a direct action of guanidine on muscle fibers, but also from artifact changes in morphology which can be produced even under satisfactory gassing incubation conditions resulting in cell injury. Curiously, the inverse relation of fibers affected *versus* time duration of Tyrode incubation seen in EDL and SOL suggests that changes were transitory and likely did not involve severe structural alterations in fibers. Based on the findings, the effect of guanidine in EDL may be related with high number of sodium channels in this muscle which interference by the drug provokes alteration of ionic conductance and osmotic disturbances in muscle fibers. This interpretation is in line with the regression in the proportion of fibers affected which passed from 21% after 15/30 min to 13% at the end of 60 mi. On the other hand, in SOL guanidine effect may involve mitochondrial effect (see below). 

The antagonistic effect of guanidine on dantrolene effect [38], a substance able to block the entry of Ca^2+^ into SR, as well as the secondary facilitation (evoked even under direct stimulation), supports the idea that guanidine has a muscular action, what was currently corroborated. The increase of cytosolic Ca^2+^ concentration (after K^+^ channel blockade) inside muscle fibers in response to membrane depolarization triggers neurotransmitter release and muscle contraction; the increase in intracellular Na^+^ results in the interruption of the change between extracellular sodium and intracellular calcium. As a result, the high intracellular Ca^2+^ concentration activates intracellular calcium-dependent proteases, which hydrolyze the components of muscle cells [22,39]. Therefore, the data indicate that, in addition to presynaptic pharmacological effects, guanidine also produces a muscle pharmacological action on the diaphragm [22,40], EDL and SOL.

Molecular characteristics of skeletal muscle types are herein considered as an attempt to explain the differences in the contractile response of PND, EDL and SOL. As premise, it is common sense that the preservation of the membrane electrochemical gradients for Na^+^, K^+^, and Ca^2+^ is vital to the muscle processes of EC coupling, regardless the muscle phenotype. Herein, we found that evoked end-plate potentials produced a membrane depolarization of diaphragm which was greater in the post-washing secondary facilitation than in the former. This peculiar effect did not find similarity to fast- and slow-twitch muscles. Why EDL and SOL do not respond that way; what are the possible causes of this?

Studies have shown that density and types of ion channels involved in neurotransmission may differ in different muscle fiber types. For instance, NMJ of fast-contracting fibers (glycolytic) have high density of Na^+^ channels, and this density is determined by the type of motor innervation [41,42,43]. Based on this, it was expected that PND and EDL muscles would present a higher density of Na^+^ channels than SOL, since they have a higher proportion of glycolytic fibers with fast contraction. As a result, PND and EDL are expected to present a larger proportion of damaged fibers than SOL muscles, which actually occurred in preparations incubated for 15 and 30 min, but not in those incubated for 60 min. Guanidine is able to cross Na^+^ channels [37,38], being prevented from returning to the extracellular compartment by the selectivity of the Na^+^ pump, which does not recognize it [44]. The accumulation of Na^+^ and Ca^2+^ into the nerve terminal, along with the guanidinium cation influx, is expected to promote osmotic fluid entry and activation of calcium-dependent endoproteases. 

Likewise, the existence of different types/proportion of K^+^ channels in all three muscles could have a role in their response to guanidine. K^+^ channels are membrane integral proteins with different types of pore-forming α-subunits; they are present ubiquitously in several types of cells where they play key roles in cell function and homeostasis; in skeletal muscle, they maintain the resting membrane potential, regulate the action potential duration and neurotransmitter release [45,46]. Recent studies provided evidences that ATP-regulated potassium (K^+^_ATP_) channel densities and channel subunit composition vary in different muscle fiber types, contributing to their differential physiological performance and determining differential pharmacological actions of drugs modulating the channel activity [47,48]. K_ATP_ channels were shown to have muscle-specific roles and are physiologically and pharmacologically regulated. They determine muscle phenotype-specific pharmacological control of electrical activity, being its density higher in fast-twitch than in slow-twitch muscles; in so doing, K_ATP_ channels play important roles in the control of contractility, protecting tissue against calcium overload (see Flagg *et al.* [48] for review). Similarly, functional and pharmacological properties of calcium-activated K^+^ channels in skeletal muscle tissue were shown to be also dependent on muscle phenotypic characteristics [49]. 

The contractile and relaxing activity (EC coupling) of muscle fibers is crucially dependent on the calcium signaling and handling apparatus; as part of the process, there are myriads of regulatory proteins, such as the main proteins (ryanodine receptor, the SR Ca^2+^ release channel; troponin, which mediates contraction through the action on Ca^2+^ interaction with myofibrils; Ca^2+^ pump, responsible for reuptaking Ca^2+^ into the SR, and calsequestrin, the Ca^2+^ storage protein in the SR) and accessory proteins (annexins, beta-actinin, calcineurin, calmodulin, calpain, myosin light chains, parvalbumin, S100 and sorcin), all of them involved in muscle response following a nerve impulse and action potential propagation. Since a range of heavy myosin isoforms may constitute muscle fiber types, the contractile properties of them vary according to their constitutive regulatory and accessory protein modulation [50,51]; thereby a series of Ca^2+^-dependent physiologic processes will be differentially modulated, including transmitter release in motor nerve endings and contraction in metabolic/physiologic distinct muscle types [27]. For instance, Zubrzycka-Gaarn *et al.* [51,52] found that the protein amount of Ca^2+^-ATPase and calsequestrin, both responsible for transporting Ca^2+^ back into the sarcoplasmic reticulum (SR), was several times lower in the slow-twitch muscle SR vesicles, and that the active Ca^2+^ transport and formation of the phosphorylated intermediate were several times lower than that observed in fast-twitch muscles. The authors suggested that the slow rate of calcium transport, found in slow-twitch muscle SR vesicles, may be related to a low content of Ca^2+^-transporting ATPase in the membrane. In addition, the impact of aging on the Ca^2+^ pump function of skeletal muscle SR, differing in soleus and gastrocnemius of rats, indicates that muscle contractile properties and SR function are muscle specific [52]. 

It is now established that the regulation of the EC coupling mechanism differs between fast-twitch and slow-twitch fibers and that there is a direct involvement of calsequestrin in the Ca^2+^ release process, given that protein represents an endogenous modulator of ryanodine receptors (SR Ca^2+^ release channels) [53,54]. Evidences have been provided that key membrane proteins involved in Ca^2+^ homeostasis detected fiber-type-specific differences in auxiliary subunits of dihydropyridine receptor, ryanodine receptor and Ca^2+^-ATPase, as well as in triad markers and various Ca^2+^-binding and ion-regulatory proteins [55]. The authors concluded that the type of motor innervation has a profound impact on the levels and isoform expression pattern of Ca^2+-^regulatory membrane proteins and reflects differences in the regulation of Ca^2+^ homeostasis and contractile response in different types of muscle.

Feldmeyer *et al.* [56] suggested that the inhibitory action of guanidinium ion on excitation-contraction coupling is due to a depression of calcium release from the SR. It is possible that the differential contractile response in all three studied muscles is also related to the population of mitochondria present in each type of muscle fiber. Since Ca^2+^ is one of the main protagonists in the genesis of the triphasic effect of guanidine, and relating it to the fact that mitochondria and SR are the main organelles involved in intracellular Ca^2+^ homeostasis, it is expected that muscle fibers with a larger number of mitochondria are the most affected by guanidine [57]. In fact, SOL muscle, which increased the number of affected cells only after 60 min of incubation (by more than twice compared to that observed at 15 and 30 min) is predominantly constituted of oxidative fibers with a larger proportion of mitochondria. It is known that mitochondria are the main organelles involved in alterations provoked by guanidine, in addition to the participation of the SR in pathophysiological processes of the drug [22,57]. 

Whether the amount of cells altered by the guanidine action was responsible for such differences is unclear. What is known is that the physiological characteristics of diaphragm [34,35,36,37] distinguish this muscle from the peripheral ones, responding differentially to endogenous or exogenous pharmacological stimuli. 

The primary and secondary facilitations caused by guanidine specifically for PND suggest that the stimulation threshold for PND to produce a maximum depolarization of the end-plate is not the same stimulation threshold for EDL and SOL muscles, *i.e.*, the threshold to cause an end-plate potential greater for PND is likely smaller than the stimulation threshold for EDL and SOL muscles. What mechanisms can account for such differences? Since diaphragm is a vital muscle, it has the ability to support adverse conditions that peripheral muscles do not have. The term “safety factor” (SF) at the NMJ is used to indicate a property of the diaphragm that maintains neuromuscular transmission operative in conditions not supported by non-vital muscles. The SF relies on both the amount of presynaptic transmitter released per nerve impulse, being greater than that required to trigger an action potential in the muscle fiber, and on elaborate postsynaptic specializations to enhance the transmitter response [58,59]. It also relies on the diaphragm, possessing a higher density of nAChR and ACh quantal contents than peripheral muscles [23]. This means that the SF makes the diaphragm more capable of amplifying the responses to depolarization, promoting the transduction process that leads to contraction than EDL and SOL muscles. Interestingly, Ermilov *et al.* [60] found that SF varies for neuromuscular transmission across muscle fiber types (even within a single muscle), being larger for type IIX or IIB fibers (fast-twitch types) than for type I or IIA fibers (slow-twitch types). As known, SOL muscle has high predominance of fiber I and IIA types, whereas diaphragm and EDL have high predominance of fiber IIX and IIB types. 

A growing interest has been directed towards the mechanism of action of guanidine and its alkyl analogs on potassium channels [20,61]. The increase of ACh release into the synaptic cleft and stimulation of neuromuscular transmission resulting from this action has profound implication in the prospect of drugs targeting neuromuscular diseases. A recent study has shown that guanidine and derivatives bind within the intracellular pore of potassium channel and disturb the hydrophobic subunit interface to stabilize a closed state of the channel [61].

## 3. Conclusions

Guanidine evoked a differential contractile response from diaphragm, *extensor digitorum longus *and *soleus* preparations. It seems unrelated to guanidine concentration, stimulation via (direct or indirect) and at some instance to the number of cells intoxicated by the drug. Instead it seems more related to intrinsic nerve-muscle mechanisms, which entail differential interaction of each muscle with the drug. Our results point two interesting issues. One issue deals with the post-blockade secondary facilitation of PND after replacement of guanidine for Tyrode solution in the incubation medium. The so-called triphasic effect, selective for PND preparation, appears to be ought to the safe factor (SF) across the NMJ that provides an over presynaptic release of ACh per nerve impulse, being greater than that required to trigger an action potential in the muscle fiber; in addition, the SF would be postsynaptically represented by high proportion of nAChR present on elaborate junctional fold specializations, typical of diaphragm. Altogether, a lower stimulation threshold would be required for PND to produce a maximum depolarization of the end-plate. A second issue is represented by the unique guanidine action on diaphragm, enhancing neurotransmission, which does not find a similar action to a slow-twitch, predominantly oxidative muscle (SOL), or fast-twitch, predominantly glycolytic muscle (EDL). Considering that the skeletal muscles are composed of different fiber types with heterogeneous density and types of ion channels, mitochondrial population, SR development and metabolic activity, it is suggested that differences in the amplitude of muscle contraction here seen in response to guanidine rely on the fine tune among ion channels *versus* variable expression of proteins involved in calcium signaling and handling. The present results reiterate the singularity of diaphragm, the largest and most important inspiratory muscle, in its response to pharmacological drugs, so well-endowed of force reserve to support the energy spent in ventilation work. This experimental design allowed imply that guanidine effect on limb and ventilator muscles differ in magnitude, depending on the muscle phenotype and duration of incubation bath. Future studies aimed at investigating the density and types of K^+^, Na^+^ and Ca^2+^ channels and the modulation of the EC coupling machinery associated with SR calcium release/reuptake cycle, mitochondrial function; and/or alterations in the action potential configuration, MEPP and quantal content, being available for the next ACh release in diaphragm, EDL and SOL can shed light on differences relative to interaction with guanidine. The present study formulates several questions to which there are no clear-cut replies; hence, the study opens a vast perspective for investigation that, when responded, will contribute relevantly to the knowledge of NMJ physiology. 

## 4. Experimental

### 4.1. Animals

Adult male Swiss mice (*Mus musculus*) of 30–40 g body weight were supplied by the Multidisciplinary Center for Biological Investigation (Cemib/Unicamp, Campinas, SP, Brazil). The animals were housed at 24 °C with free access to food (Labina, Purina, Campinas, SP, Brazil) and tap water. The experiments were done in accordance with the guidelines established by the Brazilian Society for Laboratory Animal Science (SBCAL, formerly COBEA) and approved by the University’s Ethics Committee on Experimental Animal Use (CEUA/IB/Unicamp, protocol n. 1898-1).

### 4.2. Nutritive Solution

Tyrode solution was used as a nutritive medium for muscle dissection and to provide a physiological fluid for assays on isolated preparations; the solution had the following composition [mM]: NaCl 137, KCl 2.7, CaCl_2_ 1.8, MgCl_2_ 0.49, NaHCO_3_ 11.9, NaH_2_PO_4_ 0.42, glucose 11.1. The pH was adjusted to 7.4 and the temperature was kept constant at 37 °C during twitch recordings.

### 4.3. Guanidine Solution

Guanidine chloride (organic cation salt, G4505, Sigma, St. Louis, MO, USA) was used at concentrations of 1 and 5 mM for EDL and 5 and 10 mM for all nerve-muscle preparation types evaluated.

### 4.4. Experimental Protocol

The contractile response of PND, EDL and SOL was recorded after 15, 30 and 60 min of guanidine incubation (n = 8 per time interval) under indirect stimulation, based on previous studies showing that the initial facilitation of the contractile response occurred 15 min after PND incubation, blockade occurred between 30–60 min, and the post-washing facilitation was stable 20 min after guanidine removal from incubation bath [19]. After these time-points, guanidine was removed from the bath and replaced by fresh Tyrode solution (three times), and the recording was reestablished for another approximate 20 min. Control preparations were incubated with Tyrode solution (n = 3 PND, n = 8 EDL and SOL per time interval) under the conditions used for guanidine. Furthermore, PND preparations were either pre-incubated with 4-aminopiridine (10 µg/mL) and then with 5 mM guanidine, or with 5 mM guanidine followed by 4-AP. Between the treatments, the preparation was washed (3×) for removal of the substance before addition of the other. Experiment with and without washing was done exclusively when guanidine was added before 4-AP.

### 4.5. Diaphragm Preparation

After being anesthetized with halothane inhalation (Cristália, Campinas, SP, Brazil), the mice were sacrificed by exsanguination and diaphragms were excised together with the left and right phrenic nerve trunks. The left nerve-hemidiaphragm preparation was mounted according to the method of Bülbring [61] for rat. PND preparation was suspended in a 5 mL organ bath aerated with carbogen (95% O_2_ plus 5% CO_2_) and kept under a constant 5 g/cm tension in Tyrode solution (pH 7.4, 37 °C). The preparation was indirectly stimulated using electrodes placed around the phrenic nerve (0.1 Hz, 0.2 ms, supramaximal stimuli until 3 V), delivered from a Grass S4 electronic stimulator). Isometric muscle twitch was recorded by a force displacement transducer (Load Cell BG-10 GM, Kulite Semiconductor Products Inc., NJ, USA) coupled to a physiograph (Gould Universal Amplifier, model RS 3400, Cleveland, OH, USA). The preparation was allowed to stabilize at least 20 min before guanidine (10 mM) addition.

### 4.6. EDL and SOL Preparations

Briefly, mouse isolated *extensor digitorum longus* and *soleus* muscles were removed under anesthesia. Both muscles were isolated from their distal to proximal tendon insertion, under continuous Tyrode solution bathing. The proximal tendon was fixed to the bottom of the organ bath (3 mL of Tyrode solution aerated with carbogen, 95% O_2_ plus 5% CO_2_, pH 7.4, 37 °C, and kept under a constant 0.5 g/cm tension) and the distal one was connected to an isometric transducer coupled to a physiograph. The preparations were electrically stimulated at the equatorial region of the muscles, using a platinum bipolar electrode. Stimuli were likewise maximal (until 6–8 V, 0.1 Hz frequency and 0.2 ms duration). After stabilization (at least 20 min), guanidine (10 mM for SOL and 5 and 10 mM for EDL) was added to the bath.

### 4.7. Morphological Analysis and Morphometry

At the end of the myographic recordings (15, 30 or 60 min), the muscles were fixed in Bouin's acetic solution for 24–30 hours. Diaphragm tissue samples were collected from the phrenic nerve branching region and consisted of ~5 × 8 mm and 5 × 4 mm tissue pieces. EDL and SOL muscle samples were obtained from the equatorial region, while the tendon region was discarded. Muscle fragments were dehydrated in increasing ethanol series and processed for embedding in historesin (Leica). Serial sections, 2 µm thick, were stained with toluidine blue and mounted in Entelan. The extent of muscle damage was assessed qualitatively and quantitatively (transversal section) by counting 1000 fibers (normal or damaged) in three non-overlapping randomized areas with 80 µm of distance from each other per preparation totaling 6000 fibers counted per treatment. Morphometric analyses were done in a BX51 Olympus light microscope (Tokyo, Japan) coupled to an image analyzer and two morphological patterns were established and quantified: 1) normal cells were those which appeared with no morphological abnormality in the myofibrillar organization, nucleus location, and typical polygonal profile; 2) damaged cells were those showing microvacuoles, changes in color (intensely stained), partial or total digestion of myofibrils, presence of delta-shaped lesions or visible interruptions of the fiber membrane.

### 4.8. Statistical Analysis

Each experimental protocol was repeated from three to eight times and the results reported as the mean ± S.E.M. were used for statistical comparison, using one-way analysis of variance (ANOVA) followed by Tukey’s post-hoc test, with a p-value ≤ 0.05 indicating significance.
